# Mapping Groundwater Dependent Ecosystems in California

**DOI:** 10.1371/journal.pone.0011249

**Published:** 2010-06-23

**Authors:** Jeanette Howard, Matt Merrifield

**Affiliations:** The Nature Conservancy, San Francisco, California, United States of America; University of California, United States of America

## Abstract

**Background:**

Most groundwater conservation and management efforts focus on protecting groundwater for drinking water and for other human uses with little understanding or focus on the ecosystems that depend on groundwater. However, groundwater plays an integral role in sustaining certain types of aquatic, terrestrial and coastal ecosystems, and their associated landscapes. Our aim was to illuminate the connection between groundwater and surface ecosystems by identifying and mapping the distribution of groundwater dependent ecosystems (GDEs) in California.

**Methodology/Principal Findings:**

To locate where groundwater flow sustains ecosystems we identified and mapped groundwater dependent ecosystems using a GIS. We developed an index of groundwater dependency by analyzing geospatial data for three ecosystem types that depend on groundwater: (1) springs and seeps; (2) wetlands and associated vegetation alliances; and (3) stream discharge from groundwater sources (baseflow index). Each variable was summarized at the scale of a small watershed (Hydrologic Unit Code-12; mean size = 9,570 ha; n = 4,621), and then stratified and summarized to 10 regions of relative homogeneity in terms of hydrologic, ecologic and climatic conditions. We found that groundwater dependent ecosystems are widely, although unevenly, distributed across California. Although different types of GDEs are clustered more densely in certain areas of the state, watersheds with multiple types of GDEs are found in both humid (e.g. coastal) and more arid regions. Springs are most densely concentrated in the North Coast and North Lahontan, whereas groundwater dependent wetlands and associated vegetation alliances are concentrated in the North and South Lahontan and Sacramento River hydrologic regions. The percentage of land area where stream discharge is most dependent on groundwater is found in the North Coast, Sacramento River and Tulare Lake regions. GDE clusters are located at the highest percentage in the North Coast (an area of the highest annual rainfall totals), North Lahontan (an arid, high desert climate with low annual rainfall), and Sacramento River hydrologic regions. That GDEs occur in such distinct climatic and hydrologic settings reveals the widespread distribution of these ecosystems.

**Conclusions/Significance:**

Protection and management of groundwater-dependent ecosystems are hindered by lack of information on their diversity, abundance and location. By developing a methodology that uses existing datasets to locate GDEs, this assessment addresses that knowledge gap. We report here on the application of this method across California, but believe the method can be expanded to regions where spatial data exist.

## Introduction

Only ∼1% of freshwater resources on the Earth's surface are contained within surface waters – such as rivers, lakes, and swamps. The remaining 99% is stored in either icecaps/glaciers (69%) or in groundwater (30%). Because of groundwater's accessibility and quantity, groundwater is a vital source of freshwater for human communities throughout the world [1], [2], [3].

In the U.S. and other developed countries, the value of groundwater for drinking water, irrigation, and industry is reflected in government policies that control groundwater availability and quality (e.g. U.S. EPA 2002). Some governments, including Australia [4] and European countries through The European Union (EU) Groundwater Directive (GWD Directive 2006/118/EC) [5] also now require the ecological condition of groundwater ecosystems to be considered when making policy decisions. However, in the U.S. few or no policies consider groundwater dependent ecosystems when allocating resources.

Most groundwater conservation and management efforts focus on protecting groundwater for drinking water and for other human uses with little understanding or focus on the ecosystems that depend on groundwater. The disconnect between ecological and human uses of groundwater is key as it suggests that policies and regulations that protect groundwater for human purposes may not necessarily protect groundwater dependent ecosystems (GDEs).

Although groundwater monitoring is incomplete in many parts of the world, available data suggest that groundwater supply and quality are widely threatened by over-extraction and contamination [1]. This loss and degradation are likely to increase in the future, as a result of climate-change-induced drought and human population growth, with serious consequences for both people and ecosystems [1].

Groundwater plays an integral role in sustaining certain types of aquatic, terrestrial and coastal ecosystems, and their associated landscapes, by providing inflow which maintains water levels, water temperature and chemistry required by the plants and animals they support [1]. Groundwater provides late-summer flow for many rivers and can create cool water upwelling critical for aquatic species during high temperatures, and groundwater is the only water source for springs and subterranean ecosystems which harbor a distinct and poorly understood fauna [1]. Therefore, groundwater is an important factor in maintaining the ecological integrity of some ecosystems [6], [7], [8], [9]. We define groundwater dependent ecosystems as terrestrial, aquatic, and coastal ecosystems that require access to, replenishment or benefit from, or otherwise rely on subsurface stores of water to function or persist.

In general, classifying groundwater-related ecosystems can be done by their geomorphologic setting (aquatic, terrestrial, and coastal) and associated groundwater flow mechanisms (deep or shallow) [7]. On this basis, a number of groundwater dependent ecosystem types are recognized and addressed in this paper:

- **Springs and seeps**: Discharge from relatively deep groundwater flow systems rising to form distinctive springs with associated (often unique) aquatic ecosystems (e.g. Cuatros Ciengas in the northern Mexican state of Coahuila). Springs and seeps can vary seasonally and depend on the depth and size of the groundwater resource supporting them;
- **Wetland ecosystems**: Discharge of shallow (and sometimes perched) groundwater flow (e.g. the prairie wetlands of the northern U.S. and Canada);
- **Baseflow in river systems**: Groundwater discharge varies temporally and provides dry-weather flow in river systems which is especially important in arid, semi-arid and Mediterranean climates (e.g. perennial streams in the arid to semi-arid Southwestern U.S.); and
- **Vegetation**: Phreatophytic vegetation extracts moisture directly from the water-table (e.g. oaks in Mediterranean climates – those with hot, dry summers and mild, wet winters).

To protect ecosystems that depend on groundwater a basic understanding of types and where they occur is needed. Unfortunately, in the US and many other countries, little of the relevant information is readily available at the scale of large regions (e.g. states or provinces) or entire countries [1]. To address this knowledge gap, we developed a Geographic Information System (GIS)-based method that uses existing datasets to identify where groundwater sustains surface ecosystems. Here, we report on the application of this method to the U.S. state of California to help illuminate the connection between groundwater and surface ecosystems. We analyzed readily available geospatial data at the statewide scale to identify and map the groundwater dependent ecosystems that occur in California. We compiled geospatial data for three ecosystem types that have the potential to be dependent upon groundwater: springs; groundwater dependent wetlands and associated vegetation alliances; and groundwater dependent stream channels. This effort provides a statewide index of groundwater dependency. The intent of the analysis is to provide a visualization of the biodiversity nexus of groundwater across the landscape– to better understand what biological targets are most dependent on groundwater and how they are distributed across the state. This broad-scale analysis provides a depiction of the distribution of GDEs in California and is not meant to describe groundwater processes or mechanics. We hypothesize that this type of coarse-scale accounting tool will identify GDE clusters across the state.

We anticipate the results of this study may help inform conservation of groundwater-dependent biodiversity by illuminating the extensive distribution of groundwater dependent ecosystems throughout the state.

We hope the results provide a concrete depiction of groundwater dependent ecosystems in California and “put a face” on what is to many an abstract issue. Although groundwater is only one factor in ecosystem sustainability, efforts are needed to make groundwater use and existing conservation practices more compatible.

### Regional Context: Groundwater in California

California is an important test case for developing a better understanding of GDEs at the statewide scale for two main reasons:

- The mapping and monitoring of groundwater resources within the state is inconsistent and not well developed;
- Groundwater is an unregulated, diminishing resource within the state.

**Figure 1:**
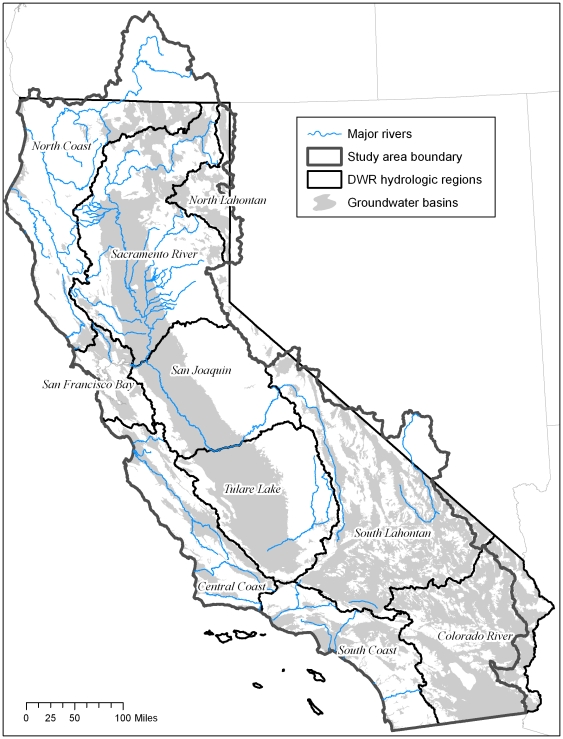
Map of study area. Map of study area including the California Department of Water Resources hydrologic boundaries and groundwater basins in California.

Groundwater is one of California's greatest natural resources, meeting 30–40 percent of California's urban and agricultural demands [10]. In 1995, the state's Department of Water Resources (DWR) estimated that 13 million Californians (40% of the state's population) used groundwater for at least a portion of their drinking water supply. Some cities, such as Fresno, Davis and Lodi rely solely on groundwater for their drinking water supply. Groundwater use has increased from an estimated 9 million acre feet in 1947, to 15 million acre feet in 2002. California's mapped 431 designated groundwater basins hold approximately 850 million acre-feet of water, only about half of which is close enough to the surface to be pumped economically [10] (Figure 1). However, these basins are just a subset of the aquifers underlying the state as not all groundwater is contained within these large, productive basins [10]. There are many other aquifers in the state that provide locally important water sources that are not within the mapped groundwater basin boundaries, and are not well understood.

For planning purposes, California has been divided by the DWR into 10 hydrologic regions which correspond to the state's major drainage areas (Figure 1). These hydrologic regions exhibit similar precipitation, runoff, geologic and tectonic conditions [11]. A review of average water year supplies from the California Water Plan [12] shows the importance and range of groundwater as a local supply for agricultural and municipal uses stratified by hydrologic region (Table 1). For example, while only 5 percent of water demand is met by groundwater in the San Francisco Bay region, over 80% of the water needs/demands of Central Coast are met by groundwater [10]. This wide range of groundwater contribution to water use is a reflection of a combination of factors including the development of external water supplies (such as water supplied to the San Francisco Bay area from the Sierra via pipelines and canals), availability of groundwater, the relative availability of water from other sources, and historical development of infrastructure and water supply practices.

**Table 1:** Annual agricultural and municipal water demands met by groundwater in California's 10 hydrologic regions [8].

Hydrologic Region	Total Demand (acre feet)	Demand met by Groundwater (acre feet)	Demand met by Groundwater (%)
Central Coast	1,263	1,045	83
Colorado River	4,467	337	8
North Coast	1,063	263	25
North Lahontan	568	157	28
Sacramento River	8,720	2,672	31
San Francisco Bay	1,353	68	5
San Joaquin River	7,361	2,195	30
South Coast	5,124	1,177	23
South Lahontan	480	239	50
Tulare Lake	10,556	4,340	41

Numbers in millions.

Despite California's heavy reliance on groundwater for human wellbeing, groundwater is a locally controlled resource, and is not regulated by the State [10]. In 1914, a system of appropriating surface water rights was created by the state through a permitting process, but groundwater was not included in that regulatory process. The regulation of groundwater has been considered at various times, however the California Legislature has repeatedly decided that groundwater management should remain a local responsibility [13]. As a result, California is the only state in the country without a comprehensive statewide groundwater management system. This has resulted in the state lacking a cohesive, dedicated monitoring network to evaluate the health of its groundwater resources.

Annual statewide overdraft is estimated by the DWR to be approximately 1.4 million acre-feet in a normal year. Most of this overdraft occurs in the San Joaquin Valley and the Central Coast [10]. Overdraft can have negative impacts on certain aquatic flora and fauna in California. For example, in the Great Basin and Mojave deserts, planned groundwater withdrawal is expected to greatly reduce spring discharge [9]. This decreased discharge is predicted to result in a reduction of areal cover of wetland vegetation and the amount of upland phreatophytic (deep rooted) vegetation by causing water table levels to drop below plant rooting depths [9]. In addition, percolation of salts to surface soils may be reduced in this same region eventually altering desert shrub cover from halophytes to nonhalophytes [9].

Overdraft can also result in saltwater intrusion into the groundwater aquifer as occurred in the Oxnard basin in the 1950s (Ventura County). There groundwater overdraft resulted in groundwater levels declining below sea level which caused seawater to intrude into fresh water aquifers [14]. To reverse the seawater intrusion process, costly recharge efforts are required to recharge the aquifers by conveying 60,000 acre feet of water through the Santa Clara River system north of the groundwater basin to spreading grounds [14].

Currently (Spring 2010), California faced a third consecutive year of drought conditions. While development of new surface water diversions and storage has slowed, new groundwater development continues at a strong pace. For example, in Kings County, newspaper articles report that local well drilling businesses are busier than ever, as water deliveries from the Sacramento Delta to farmers in the Westlands Water District have decreased. In addition, growers are drilling deeper, as far as 2,000 feet in some cases, to access diminished aquifers with low salt levels. In May 2009 the Butte County Department of Water and Resource Conservation reported that due to lack of rain and groundwater withdrawals, 37 of the 81 wells monitored were at an “alert stage” requiring irrigation coordination in the county. Since 2006 the water levels of the aquifer in the San Joaquin basin (Tulare County) have dropped 50 feet resulting in some existing pumps no longer reaching far enough to bring water to the surface.

## Methods

### Study Area

The geographic study boundary is confined to watersheds that flow into California's boundaries and omits those watersheds that flow into adjacent states (such as the Great Basin streams). This boundary corresponds to the USGS National Hydrologic Database (NHD) Region 18 [15] (Figure 1).

Geospatial data were compiled to create three variables to represent ecosystem dependence on groundwater:

1. density of springs and seeps at the HUC12 scale;
2. density of groundwater dependent wetlands and associated vegetation alliances (hereafter referred to as wetlands) at the HUC12 scale; and
3. percent of discharge from groundwater (baseflow) at the HUC12 scale.

**Figure 2:**
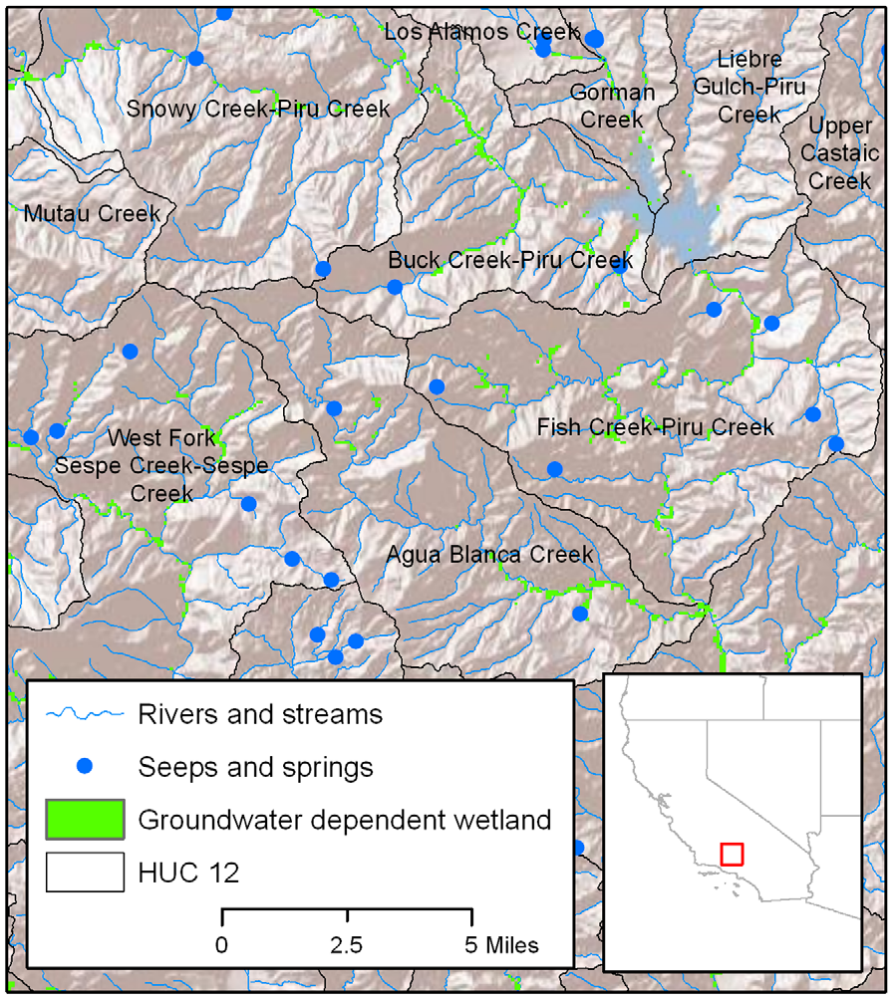
Scale of analyses – HUC12 units. Examples of HUC12 analyses units in the Southern California hydrologic region.

Each variable was summed to finest USGS hydrologic unit scale - the 12^th^ level Hydrologic Units of the USGS (referred to as HUC12). There are 4,621 of these units in the study area with a mean size of 9,570 hectares (Figure 2). To make biogeographic comparisons, the fine-scale HUC12 unit variables were binned and summarized at the DWR hydrologic basin scale, as these are a commonly used geographical subdivision for water resource management in California, and are areas of relative homogeneity in terms of hydrologic, ecologic and climatic conditions.

### Index of Groundwater Dependency

An index of groundwater dependency was developed by mapping and ranking three ecosystem types – springs, wetlands streams as follows:

#### 1) Seeps and springs

Seeps and springs were extracted from the National Hydrography Dataset Plus [16] database and assigned to each HUC12 unit. Springs are mapped as point features in the NHD Plus dataset and therefore do not contain areal extent. To avoid scoring larger HUC12 units with higher scores (likely that larger units would contain more springs) density of springs was therefore calculated as the number of springs and seeps per hectare. The raw density values were categorically scored (from 0–4) for each HUC12 using distribution quartiles (Table 2). Note that a score of 0 means that there were no springs in the HUC12 unit.

**Table 2:** Groundwater dependent ecosystems and the variables used to represent those ecosystem types.

Ecosystem	Variable	Score
Springs and Seeps	Number per area HUC12	1 = (.001–0.247 springs/1,000ha)2 = (0.21–0.44 springs/1,000ha)3 = (0.441–0.94 springs/1,000ha)4 = 0.942–11.8 springs/1,000 ha)
Wetlands and Vegetation	Areal extent per area of HUC12	1 = (.0475–1.97 ha/1,000ha)2 = (1.99–7.842 ha/1,000ha)3 = (7.844–24.808/1,000ha)4 = 24.81–81. 080 ha/1,000 ha
Baseflow Index	Percent of discharge from groundwater	1 = 0.4–33.9%2 = 33.95–51.3%3 = 51.35–65.38%4 = 66.33–96.7%

Variables were created to account for the relative amount of each ecosystem type and ranked according to standardized scores for each.

It is important to note that the seeps and springs database does not contain information on the amount of flow emanating from the seeps/springs. Because we are considering springs in this study as habitat rather than discharge (water supply), we believe density of springs is sufficient in illuminating springs as a groundwater dependent variable.

#### 2) Groundwater dependent wetlands and associated vegetation alliances

To locate where groundwater flow sustains wetlands, we identified and mapped GDEs using the best available data. To conduct this assessment across the entire state, we had to rely on incomplete datasets and to make assumptions in data interpretation. We did this in two steps. First, we developed **one** composite spatial layer of wetlands and groundwater dependent vegetation alliances from various sources including but not limited to the U.S. Forest Service vegetation mapping effort known as CALVEG [17], the Multi-Resolution Land Characteristics (MRLC) Consortium's National Land Cover Dataset (NLCD) [18], and the Fish and Wildlife Service's National Wetlands Inventory [19]. Because none of these mapping efforts are complete for the entire state, we developed a composite dataset using the best available data. For the full list of data sources and wetland types and vegetation included as being groundwater dependent see Supporting Information (Text S1). The composite layer mapped all wetland and vegetation types that may have some level of groundwater dependence as determined from the data source's metadata and consultation with ecologists familiar with the specific ecosystems. These wetlands were mapped as polygon features in ArcMap. Each polygon was assigned as a wetland type or vegetation alliance as listed in the Supporting Information. Estuarine systems and lake margins were specifically omitted as there was conflicting views of whether these systems could be groundwater dependent.

Second, to omit wetlands that may not be groundwater dependent, we developed criteria for wetland inclusion in the spatial database. Although springs are groundwater dependent regardless of location, the groundwater dependence of wetlands is a function of their hydrological, geological and climatic setting (Brown et al. 2010).

Because groundwater dependent wetlands are defined by hydric or partially hydric soils [20], [21] we intersected the composite geospatial polygons with soils that contained “hydric” or “partially hydric” components from NRCS STATSGO2 [22]. We believe this step provided a filter by which surface water dependent wetlands could be removed from the database. In reviewing the data, we note that vernal pools a type of surface water wetlands were removed from the spatial data using by incorporate this step.

A total of 1,568,609 ha of wetlands were derived from the database before filtering with hydric soils. After filtering, a total of 1,046,983 ha of wetlands were included in the analysis (522,625 ha were omitted).

Resultant polygons of groundwater dependent wetlands were assigned to HUC12 units and density calculated as area of groundwater dependent wetlands per hectare. Density values were then categorically scored (from 0–4) for each HUC12 using distribution quartiles (Table 2). Note that a score of 0 means that there were no groundwater dependent wetlands in the HUC12 unit.

#### 3) Groundwater dependent streams

To develop an index of groundwater dependent streams we used the NHD 24,000- scale data set for all of California and assigned baseflow to stream segments based on U.S. Geological Survey data [23]. Base flow is the component of the streamflow that can be attributed to groundwater discharge into streams.

We assigned a baseflow index (BFI) (defined as the ratio of baseflow to total flow in a stream) to each HUC12 in the study area. We did this using BFI data from the U.S. Geological Survey [23]. The BFI calculation implements a deterministic procedure developed by the British Institute of Hydrology [24]. The method combines a local minimums approach with a recession slope test. The program estimates the annual base-flow volume of unregulated rivers and streams and computes an annual base-flow index for multiple years of data at one or more gage sites. The USGS acknowledges that the method may not yield the true base flow as might be determined by a more sophisticated analysis, however, has found the index to be consistent and indicative of base flow.

We assigned BFI values to each HUC12 unit using the following logic:

1. For all HUC12 units with a USGS stream gage present somewhere in the watershed, BFI values were assigned from gage data [23]. In the event of multiple stream gages in the watershed, an average value was assigned.
2. For all HUC12 units with streams and no stream gages present, we assigned BFI using interpolated values from a 1-km raster dataset for the conterminous U.S. estimated from stream gages [23], [25], [26]. (3) For HUC12 units with no streams or stream gauges we assigned a BFI value of zero.

Using these methods all HUC12 units were assigned the best estimate of baseflow for streams within the HUC boundaries and thus dependency on groundwater. Raw BFI values were categorically scored using distribution quartiles (Table 2).

#### Index calculation

Using the three variables discussed above, we developed an index of ecosystem groundwater dependency by summing the values of the three variables (springs, wetlands and rivers) for each HUC12 unit and mapping across the study area. In this way an index of groundwater dependency was developed that ranged from 0 to 12. The lowest ranking HUC12 unit could receive is 0 if there are no springs, no groundwater dependent wetlands and no stream reaches with a baseflow component. Alternatively, the highest ranking a HUC12 unit could be assigned is a 12 if unit contained the highest classes of springs, groundwater dependent wetlands and baseflow index.

Based on quartiles, HUCs were ranked as follows:

None = 0
Very Low = 1–3
Low = 4–5
Medium = 6–7
High = 8–12

## Results

We identified and mapped the types and locations of the three groundwater variables (springs, groundwater dependent wetlands, and baseflow index) and scaled the results to 4,621 HUC12 units in the state. As stated earlier, to make biogeographic comparisons, the HUC12 unit variables were binned and summarized at the DWR hydrologic basin scale.

### Springs

**Figure 3:**
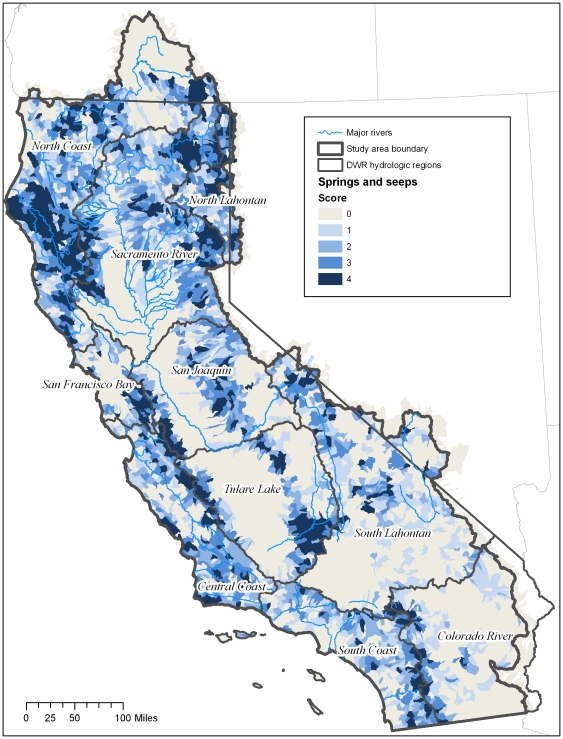
Map of density of springs in California. Map represents of density of springs per HUC12 unit. HUCs were ranked as follows based on quartile distribution: 1 = (.001–0.247 springs/1,000ha); 2 = (0.21–0.44 springs/1,000ha); 3 = (0.441–0.94 springs/1,000ha); 4 = 0.942–11.8 springs/1,000 ha).

**Figure 4 pone-0011249-g004:**
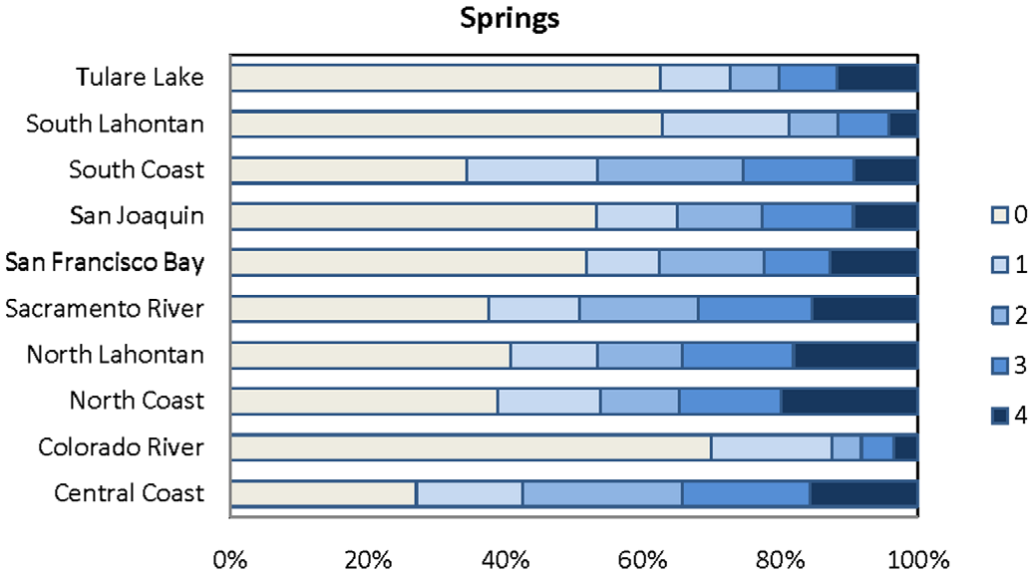
Percentage of springs per region. Percent of HUC12 units ranking 1–4 for springs per hydrologic region. 0 = no springs. See Table 2 for definition of scores.

Seeps and springs occur in 50% of HUC12 watersheds (n = 2,370) distributed throughout California (Figure 3). The North Coast hydrologic region has the greatest occurrence of springs (n = 3,604) occurring in 61% (n = 459) of the 752 HUC12 units in that region. The San Francisco Bay Area hydrologic region has the lowest number of springs (n = 347) occurring in 50% (n = 70) of the 129 HUC12 units (Figure 4). In terms of percentage, the Central Coast region has the greatest number of HUCs containing springs at 69%. The Colorado has the lowest with only 31 percent of the HUC12 units in that region containing springs.

The number of springs per 1,000 hectares is variable at the state and regional scale. For example, across the state the mean number of springs per 1,000 hectares is 0.7. The North Coast region has the greatest number of springs per area with a mean of 0.91 per 1,000 ha; the Colorado River hydrologic region has the lowest with a mean value of 0.44 per 1,000 ha.

At the subwatershed scale, the HUC unit with the highest density of springs is located in the Central Coast, where 67 springs occur in a 13,962-ha HUC unit; the lowest density is found in the South Coast hydro unit where just 1 spring was mapped in a 40,455-haHUC unit.

Based on the quartile distributions, the HUCs in each ecoregion were given a score from 1–4 (See Methods) (Table 2). The North Coast, North Lahontan and Tulare Lake regions had the greatest percentage of HUCs with scores of 4 for density of springs – over 30% in each region; the Colorado River, South Coast and South Lahontan had the lowest percentage with less than 15% of HUCs scoring 4s (Figure 3 and Figure 4).

In terms of land area, the greatest number of HUCs with a rank of 4 is found in the North Coast and North Lahontan where 20% and 18% of the land area, respectively, contain between 1–11 springs per 1,000 hectares. The percentage of the land area without springs is found in the Colorado region where 70% of the land area have no springs; in the San Joaquin where 53% have no springs, South Lahontan 63% and Tulare (62%).

### Groundwater dependent wetlands

**Figure 5 pone-0011249-g005:**
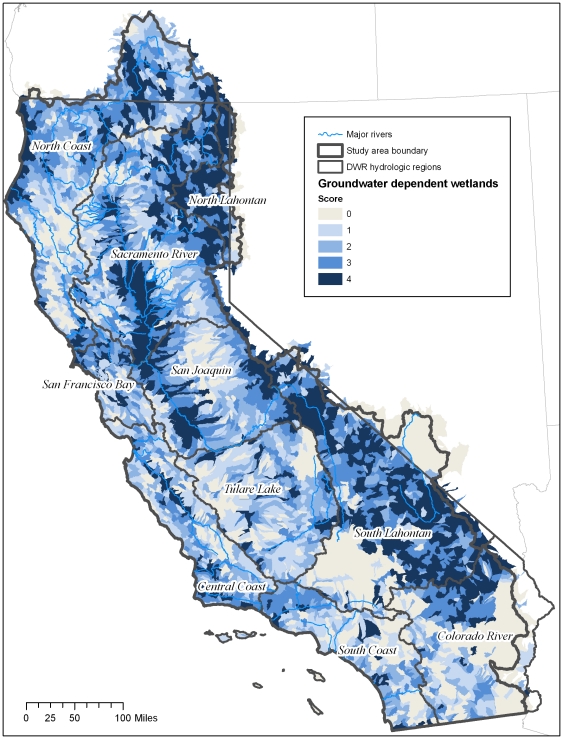
Map of density of groundwater dependent wetlands and vegetation alliances in California. Map represents of density of groundwater dependent wetlands and vegetation alliances per HUC12 unit. HUCs were ranked as quartiles as follows: 1 = (.0475–1.97 ha/1,000ha); 2 = (1.99–7.842 ha/1,000ha); 3 = (7.844–24.808/1,000ha); 4 = 24.81–81. 080 ha/1,000 ha.

**Figure 6 pone-0011249-g006:**
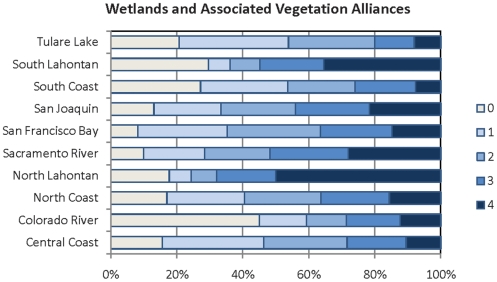
Percentage of groundwater dependent wetlands per region. Percent of HUC12 units ranking 1–4 for groundwater dependent wetlands and vegetation alliances per hydrologic region. 0 = none. See Table 2 for definition of scores.

Groundwater dependent wetlands are distributed in 76% (n = 3,526 of HUC12 units in California. The greatest cluster of HUC12 units with a presence of groundwater dependent wetlands is found in the San Francisco Bay hydrologic region where 88% of the HUC12 units in the region contain this groundwater dependent variable (Figure 5). The Colorado River region is the sparsest in terms of the number of HUCs with groundwater dependent wetlands, where only 49% of the HUC units in that region contained groundwater dependent wetlands (Figure 6).

The areal extent of groundwater dependent wetlands ranged widely throughout the state – with groundwater dependent wetlands ranging from <1 hectare of wetlands per 1,000 hectares to 810 ha/1,000 ha. As can be seen in Figure 5, the greatest density of groundwater dependent wetlands is found in the North Lahontan region where groundwater dependent wetlands average 80 hectares per 1,000 hectares of HUC12 units. The Central Coast and Tulare Lake had the lowest densities with a mean of 10.3 and 10.6 ha per 1,000 ha, respectively.

The North Lahontan and South Lahontan Lake regions had the greatest percentage of HUCs with scores of 3 and 4 totaling 81% and 76%, respectively. In terms of land area, HUCs with a rank of 4 total 50% of the area in the North Lahontan, 35% of the South Lahontan and 28% of the Sacramento River regions (Figures 5 and 6). Although the South Lahontan region has a high percentage of area ranking in the 75^th^ to 100^th^ percentile, it also has a high percentage of land area (30%) without groundwater dependent wetlands. Other regions with high percentages of land without groundwater dependent wetlands are the Colorado (45%), and Tulare (21%)

### Groundwater dependent streams

**Figure 7 pone-0011249-g007:**
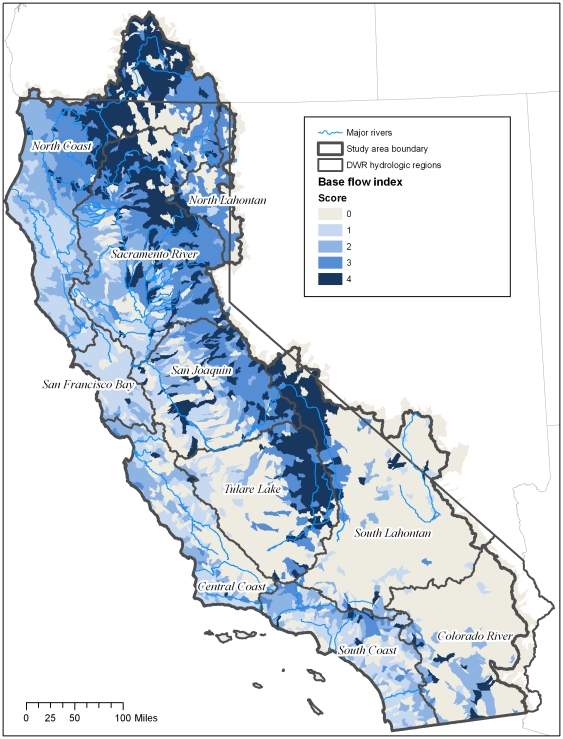
Percent of stream discharge dependent on groundwater. Map represents the percent of stream discharge composed of groundwater (baseflow index) per HUC12 unit. HUCs were ranked as quartiles as follows: 1 = 0.4–33.9%; 2 = 33.95–51.3%; 3 = 51.35–65.38%; 4 = 66.33–96.7%.

**Figure 8 pone-0011249-g008:**
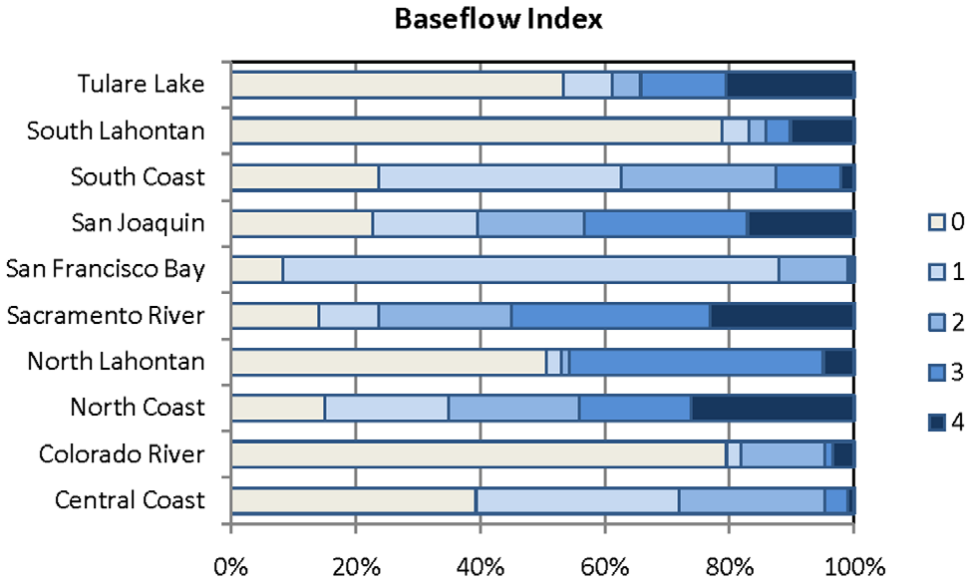
Baseflow index per region. Percent of HUC12 units ranking 1–4 for baseflow index. 0 = none. See Table 2 for definition of scores.

A total of 2,716 HUC12 units (59%) contain reaches of rivers with a baseflow index and those were included in our analysis (Figures 7 and 8). The mean baseflow of reaches by HUC unit ranged from 27 percent (San Francisco Bay) to 60 percent (North Lahontan) of the total annual stream flow. Mean baseflow per hydrologic region was greatest in the North Coast, North Lahontan, Sacramento, San Joaquin, South Lahontan and Tulare Lake hydrologic regions where >50% of the total streamflow is attributed to groundwater.

The North Lahontan had the greatest percentage of HUCs with scores of 3 and 4 (50^th^–100 percentile) at 94 percent; the San Francisco region had the lowest with just 2 percent of the HUCs scoring 3 and 4 (Figure 8).

In terms of land area, HUCs with a rank of 4 (75^th^–100 percentile) total 26% of the land area in the North Coast, 23% of the land are in the Sacramento River and 20% of the Tulare regions.

### Index of groundwater dependency

**Figure 9 pone-0011249-g009:**
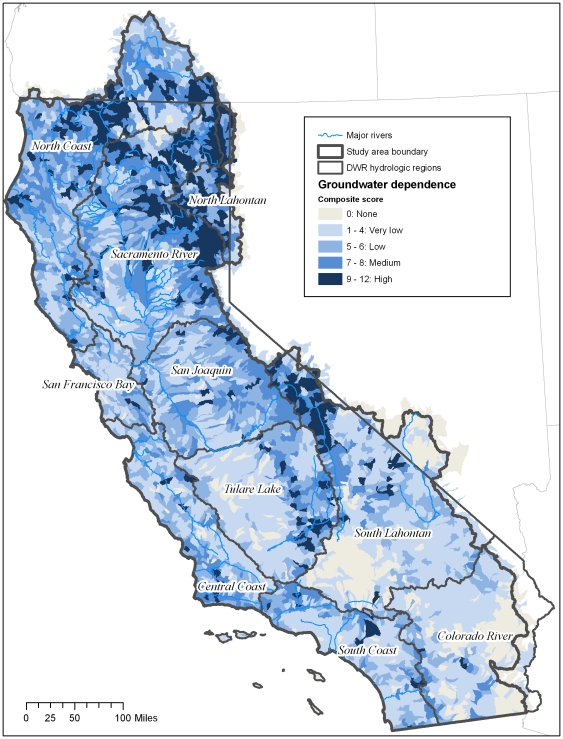
Groundwater dependence index. Map of index of groundwater dependence at the HUC12 scale in California. The index is the sum of groundwater dependent variables (springs, groundwater dependent wetlands and associated vegetation alliances and baseflow index). Based on quartile distribution of the sum, HUCs were ranked as follows: 0 = no groundwater dependent ecosystems; 1–3 = Very Low; 4–5 = Low; 6–7 = Medium; 8–12 = High.

**Figure 10:**
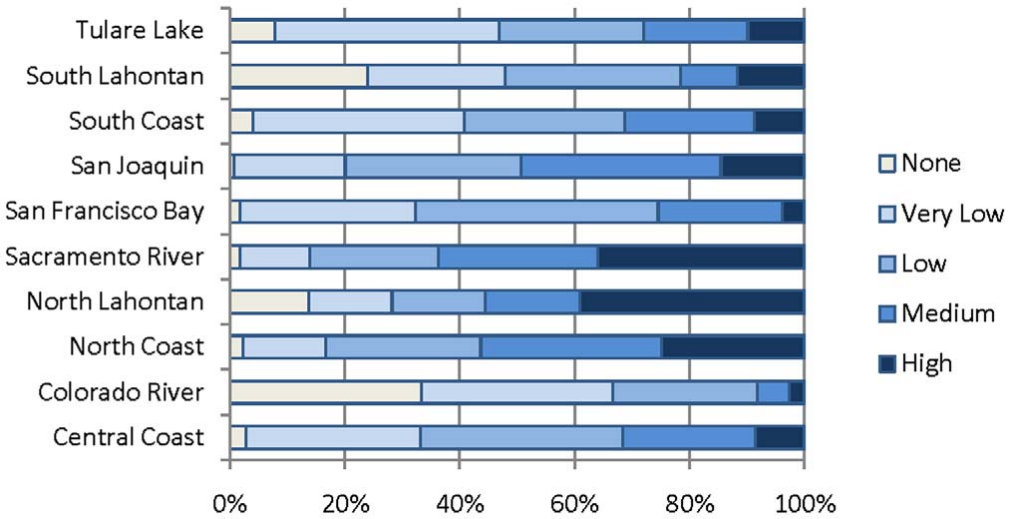
Percentage of groundwater dependence per region. Percent of HUC12 units per hydrologic region ranking very low, low, medium and high based on calculate index of groundwater dependency.

A total of 493 (11%) of the HUC12 units do not have any groundwater dependent ecosystems according to our analysis (index score = 0) (Table 3, Figures 9 and 10). These HUCs are clustered in the Colorado and South Lahontan regions where 38 and 24 percent of the HUC units, respectively, have no groundwater dependent ecosystems.

**Table 3:** Breakdown of the index of groundwater dependence rankings per hydrologic region.

Hydrologic Region	Total HUC12 units (#)	No groundwater dependency (#HUCs)	Very Low (# HUCs)	Low (#HUCs)	Medium (#HUCs)	High (#HUCs)
**Central Coast**	339	14	108	111	77	29
**Colorado River**	442	168	131	109	24	10
**North Coast**	752	20	111	209	236	176
**North Lahontan**	180	22	28	31	31	68
**Sacramento River**	802	18	105	181	227	271
**San Francisco Bay**	129	3	38	52	31	5
**San Joaquin**	429	5	89	135	143	57
**South Coast**	300	15	114	82	67	22
**South Lahontan**	790	192	197	240	73	88
**Tulare Lake**	458	36	152	136	88	46
**TOTAL**	**4621**	**493**	**1073**	**1286**	**997**	**772**

The index was developed by summing the rankings of springs, groundwater dependent wetlands and baseflow index of HUC12 units in each hydrologic region. The index ranges from 0 (no groundwater dependence) to 12 (highest score of 4 for the three variables). Rankings were defined for each HUC as follows: None = 0; Very Low = 1–3; Low = 4–5; Medium = 6–7; High = 8–12.

A total of 772 HUC12 units (14%) ranked as high (8–12) and are distributed throughout all of the hydrologic regions (Table 3, Figures 9 and 10). A total of 1,073 HUC12 units (23%) ranked as very low (1–3) (Table 3, Figures 9 and 10). The greatest percentage of HUCs ranking high is found in the North Lahontan (38 percent) and Sacramento River (34 percent)(Figures 9 and 10). HUC12 units with the lowest rankings (1–3) were concentrated in the South Lahontan and Colorado River regions where >20% and >30% of the HUCs, respectively, ranked very low.

In terms of land area the North Coast at 25%, North Lahontan at 39% and Sacramento at 36% had the greatest percentage of land with high groundwater dependence index (Score = 8–12). In contrast, the following regions had little land area ranking high: Central Coast (9%), Colorado River (3%), San Francisco Bay (4%), South Coast (9%), and Tulare Lake (10%). Regions with the greatest land area ranking as none (0) or very low (1–3) are the Colorado (67%), South Coast (48%) and Tulare (47%) (Figures 9 and 10).

## Discussion

All three types of groundwater-dependent ecosystems studied here (springs, groundwater dependent wetlands, and rivers) are widely, although unevenly, distributed across California (Figures 3–10). Although different types of GDEs are clustered more densely in certain areas of the state, watersheds with multiple types of GDEs are found in both humid (e.g. coastal) and more arid regions. Springs are most densely concentrated (high percentage of land area ranking 4) at the HUC12 scale in the North Coast and North Lahontan, whereas groundwater dependent wetlands and associated vegetation alliances are concentrated in the North and South Lahontan and Sacramento River hydrologic regions. The percentage of land area where stream discharge is most dependent on groundwater is found in the North Coast, Sacramento River and Tulare Lake regions.

Concentrations of GDE clusters (all three types) are located at the highest percentage (in terms of land area) in the North Coast, North Lahontan, and Sacramento River hydrologic regions, three distinct hydrologic and climatic regions. The highest yearly rainfall totals in California fall in the North Coast hydrologic region with areas near the Oregon border receiving ∼5,100 mm [11]. This contrasts sharply to the North Lahontan where much of the region is chronically short of water due to the arid, high desert climate, where annual precipitation can be as low as 100 mm. The Sacramento River region is characterized by strong orographic influences of the Sierra Nevada which high yearly precipitation totals (∼1,000mm), 50% falling as snow [11]. That GDEs occur in such distinct climatic and hydrologic settings reveals the widespread distribution of these ecosystems.

One potential result of this analysis is our ability to compare where groundwater is ecologically important with where it is important for human uses (Table 1). An initial assessment of our study, suggests that areas of the state with the greatest water demand correspond to areas with high concentrations of GDEs. For example, the Sacramento hydrologic region contains high concentrations of GDE clusters, and also is an area heavily reliant on groundwater withdrawals to meet urban, agricultural and industrial demands [10]. Water demands in the Sacramento region total 8.7 million acre feet, 31% of which is met by groundwater (Table 1).

In the Tulare Lake region, 62% of the land area contains no springs, and 21% of the land area contains no groundwater wetlands. However, in this region 20 percent of the land area is ranked as 4 for baseflow index – meaning that between 66–100% of stream discharge on 20% of the land area, comes from groundwater, making groundwater an important component of the stream ecosystem. Groundwater here is also important to both urban and agricultural uses, accounting for 41 percent of the region's total annual supply of 10 million acre-feet of water, and 35 percent of all groundwater use in the state [10]. Extensive groundwater recharge programs are in place in the region for future use and water banking transfer programs [10].

Groundwater development, until recently, has supplemented an abundant surface water supply [10]. However, with changing, environmental laws and requirements, and consecutive drought years, the balance is shifting to a greater reliance on groundwater [10]. The disconnect between ecological and human uses of groundwater is important, because it suggests that policies that protect groundwater for human uses may not necessarily protect GDEs. To protect groundwater resources, it is critical that we begin to manage water in a way that is more inclusive of all users, including ecosystems and species.

Because groundwater-dependent ecosystems can be affected by offsite activities that alter the hydrologic cycle [28], [29], [30], a better understanding is also needed of threats – including the threat of incremental flow reductions that might result from groundwater pumping. A finer-scale analysis is necessary to understand how groundwater extraction may affect subsurface flow paths and other groundwater processes, and in turn surface water processes.

Results of this study may help inform conservation of groundwater-dependent biodiversity by illuminating the extensive distribution of GDEs throughout the state. Areas of the state with high groundwater dependency could be the focus of future analyses to investigate the potential threats to those ecosystems by groundwater withdrawal. For example, from this analysis, we could choose specific variables – such as springs – upon which to base conservation strategies, or focus on GDE clusters. Spring ecosystems are one of several groundwater dependent ecosystems that increasingly are being affected worldwide by local and regional groundwater withdrawals [31], [32]. Potential conservation strategies could involve identifying spring ecosystems that provide critical habitat for endemic and threatened species and developing a conservation plans that provide functional protection of the diverse and rare spring ecosystems.

In summary, protection and management of groundwater-dependence ecosystems are potentially hindered by lack of information on their diversity, abundance and location. By developing a methodology that uses existing datasets to locate GDEs, this assessment addresses that knowledge gap. We report here on the application of this method across California, but believe the method can be expanded to regions where spatial data exist.

It is hoped that this analysis will help identify areas where future conservation efforts can be pursued, and shape additional scientific studies to better understand groundwater processes and the links between groundwater and aquatic ecosystems. While this study does not seek to address groundwater management in the state, we hope the results provide a concrete depiction of groundwater dependent ecosystems in California and “put a face” on what is to many an abstract issue.

## Supporting Information

Text S1 Datasets and variables used to create the composite layer of groundwater dependent wetlands and vegetation.
(0.04 MB DOC)
