# Supplementary material for: Mapping Groundwater Dependent Ecosystems in California
Source: PLoS One. 2010 Jun 23;5(6):e11249. doi: 10.1371/journal.pone.0011249 (PMC2890404; doi:10.1371/journal.pone.0011249)
Supplement: Text S1 — Datasets and variables used to create the composite layer of groundwater dependent wetlands and vegetation. (0.04 MB DOC) [file pone.0011249.s001.doc]

SUPPORTING INFORMATION

Datasets and variables used to create the composite layer of groundwater dependent wetlands and vegetation.

1. **California Department of Forestry and Fire Protection compilation of vegetation data (2001). (**[http://frap.cdf.ca.gov](http://frap.cdf.ca.gov/).)

Types included:

- 1. Desert Riparian
  2. Desert Wash
  3. Freshwater Emergent Wetland
  4. Alkali Desert Scrub
  5. Desert Riparian
  6. Palm Oasis
  7. Riverine
  8. Wet Meadow
  9. Lacustrine

1. **Central Mojave Desert Vegetation Map (2002). (**<http://www.mojavedata.gov/datasets.php?&qclass=veg> )

Note: These data were developed as part of the Department of Defense Legacy funded Mojave Desert Ecosystem Program.

Originator: Kathryn Thomas

Types included:

- Alkali Meadow/Sink
- High Elevation Wash System
- Low Elevation Wash System
- Mesquite
- Mid Elevation Wash System
- Saltbush

1. **U.S. Fish and Wildlife Service National Wetlands Inventory** (<http://www.fws.gov/wetlands/>)

Types included:

- Freshwater Emergent Wetland
- Freshwater Forested/Shrub Wetland
- Riverine

1. California Department of Fish and Game, California Natural Diversity Database (<http://www.dfg.ca.gov/biogeodata/>)

Types included:

- Alkali meadow
- Alkali seep
- Coastal and Valley Freshwater Marsh
- Desert Fan Palm Oasis Woodland
- Fen
- Great Basin Desert Spring Outflow

1. **U.S. Environmental Protection Agency, National Land Cover Data (2006) (**<http://www.epa.gov/mrlc/nlcd-2006.html>)

Types included:

- Woody Wetlands
- Emergent Herbaceous Wetlands

1. **USDA U.S. Forest Service CALVEG** (http://www.fs.fed.us/r5/rsl/)

Types included:

- Alkaline mixed scrub
- Arrow weed
- Baccharis (riparian)
- Black cottonwood
- CA sycamore
- Catclaw
- Cheesebush
- Cottonwood-alder
- Desert mixed wash scrub alliance
- Desert Willow Alliance
- Fan palm
- Fremont cottonwood
- Mesquite
- Mixed alkaline grass
- Palo Verde
- Pickleweed-cordgrass
- Saltbush
- Scale broom
- Shrub willow
- Smoketree
- Tule cattail
- Wet meadows
- Willow
- Willow-alder
- Willow-aspen
- Willow-scrub
